# Preoperative Clinical and Computed Tomography (CT)-Based Nomogram to Predict Oncologic Outcomes in Patients with Pancreatic Head Cancer Resected with Curative Intent: A Retrospective Study

**DOI:** 10.3390/jcm8101749

**Published:** 2019-10-21

**Authors:** Shin Hye Hwang, Ha Yan Kim, Eun Ju Lee, Ho Kyoung Hwang, Mi-Suk Park, Myeong-jin Kim, Woo Jung Lee, Yong Eun Chung, Chang Moo Kang

**Affiliations:** 1 Department of Radiology, National Health Insurance Service Ilsan Hospital, Goyang 410-719, Korea; pleiades5@nhimc.or.kr; 2 Department of Biomedical Systems Informatics, Biostatistics Collaboration Unit and Yonsei University College of Medicine, Seoul 03722, Korea; hykim1213@yuhs.ac (H.Y.K.); eunjulee12@naver.com (E.J.L.); 3 Division of HBP Surgery, Department of Surgery, Yonsei University College of Medicine, Seoul 03722, Korea; drhhk@yuhs.ac (H.K.H.); wjlee@yuhs.ac (W.J.L.); 4 Department of Radiology, Severance Hospital, Yonsei University College of Medicine, Seoul 03722, Korea; radpms@yuhs.ac (M.-S.P.); kimnex@yuhs.ac (M.-j.K.)

**Keywords:** pancreatic cancer, pancreatic head cancer, nomogram, computed tomography (CT), prognosis

## Abstract

Background: Currently, proposed nomograms are mainly based on post-operative histopathology. The purpose of this study was to identify preoperative computed tomography (CT) and clinical information that allow prediction of disease-free (DFS) and overall survival (OS) of patients surgically treated for pancreatic head cancer. Methods: A total of 136 patients who underwent curative-intent surgery were retrospectively reviewed. Based on results from multivariate Cox regression analysis, a prediction model was constructed with preoperative CT features and clinical information. Overall performance of the nomogram was calculated by Harrell’s C-index. Results: Symptoms at diagnosis, preoperative serum CA 19-9 ≥ 34 U/mL, and four imaging features (necrosis (DFS, *P* = 0.066; OS, *P* = 0.002), possible venous invasion (DFS, *P* = 0.150, OS, *P* = 0.055), suspected metastatic regional lymph node (DFS, *P* = 0.001; OS, *P* = 0.099), and associated pancreatitis or pseudocyst (DFS, *P* = 0.013; OS, *P* = 0.041)) were included to build the nomogram. The c-statistics for the discrimination power of the proposed nomogram was 0.6496 for DFS and 0.6746 for OS. Conclusion: A nomogram derived from preoperative CT and clinical information could estimate the risk of recurrence and all-cause death after curative-intent surgery for radiologically resectable pancreatic head cancer.

## 1. Introduction

Pancreatic cancer is the fourth leading cause of cancer-related mortality [1]. Because it tends to be detected in advanced stages, only 20% of newly diagnosed patients are thought to be surgical candidates [2]. The suspected invasion of major vessels around the pancreas, evaluated on computed tomography (CT) or magnetic resonance imaging (MRI), is the key feature to deciding resectability [3,4]. Recently, the possibility of surgical resectability has expanded, with advances in imaging and surgical techniques [5]. However, the oncologic outcomes of resected pancreatic cancer are still disappointing. The one- and five-year survival rate is approximately 60–77.8% and 20–31.3%, respectively [6,7,8], and reported median overall survival is 17–22.8 months after macroscopically complete removal of cancer. Approximately half of patients who undergo curative-intent surgery for pancreatic cancer experience recurrence within a year [8,9]. Moreover, pancreatectomy is a high-risk procedure. Perioperative mortality has declined to less than 3–4% over the past decade, owing to the increased experience of dedicated pancreatic surgeons who mostly work at specialized centers [10]. However, perioperative morbidity still remains high.

Considering the perioperative morbidity and early recurrence after standard surgical treatment and improved results of neoadjuvant therapy with the introduction of new chemotherapeutic agents such as NAB-paclitaxel (Abraxane^®^; Abraxis BioScience LLC, Summit, NJ, USA) in addition to the FOLFIRINOX (combination of folinic acid, fluorouracil, irinotecan, and oxaliplatin) regimen [11,12], appropriate patient selection is crucial to achieve the best surgical and oncologic outcomes. With the exception of several clinical factors reported to be related with the survival of pancreatic cancer patients such as age [13], gender [13], performance status [14], or serum CA 19-9 [15], generally accepted prognostic factors are mainly related to postoperative histopathologic examinations [16,17]. A prognostic nomogram, previously developed with data from a large population and externally validated, was also based on postoperative histopathology [18]. If we can predict the clinical course of a patient after curative-intent surgery based on preoperatively identified information, it can help surgeons select optimal surgical candidates and choose management approaches reflecting individual patient preference and expected quality of life. 

Hence, the purpose of this study was to identify preoperative clinical and radiologic parameters to predict one- and five-year disease-free survival (DFS) and overall survival (OS) in patients who underwent curative-intent pancreatectomy for pancreatic cancer without neoadjuvant therapy.

## 2. Materials and Methods

### 2.1. Study Population and Clinical Information

The institutional review board of our institution approved this retrospective study and waived the requirement for informed consent. One hundred and fifty-five patients who underwent surgical treatment aiming to cure pancreatic head or neck adenocarcinoma without neoadjuvant therapy were registered in the surgical database of our institution from February 2005 to December 2016. Seventeen patients were excluded from analysis because the quality of their preoperative CT images was not enough for evaluation. Two patients, who expired within 30 days after surgery, were excluded to remove cases of perioperative mortality. Therefore, the final study population included 136 patients. The following clinical information, laboratory data, and pathology reports were collected prospectively as part of routine practice by a clinical research nurse: Gender, age, body mass index (BMI), presence of symptoms at diagnosis, serum CA 19-9, serum total bilirubin, serum albumin, prognostic nutritional index (10 × serum albumin (g/dL) + 0.005 × total lymphocyte count (/mm^3^)) [19], type of surgery, surgical resection of major vessels or adjacent organs, adjuvant chemotherapy, pathologic tumor size, histologic differentiation, pathologic T stage, presence of lymph node (LN) metastasis, LN ratio (total number of metastatic LNs divided by total number of retrieved LNs), microscopic perineural invasion, lymphovascular invasion, involvement of resection margins, presence of event (recurrence or death), and time to event.

### 2.2. CT Imaging

Preoperative CT was performed using various machines and protocols with intravenous contrast agents. If there was at least one portal venous phase (PVP) CT of acceptable quality, it was used for analysis. If multiple phases of acceptable quality were available, all CT images were reviewed in the order of PVP, pre-contrast, and arterial phase (AP).

### 2.3. Image Analysis

All CT images were retrospectively reviewed using a Picture Archiving and Communication System (Centricity, Version 4.0, GE Healthcare, Barrington, IL, USA). Two board-certified abdominal radiologists (S.H.H. and Y.E.C., with 2 and 11 years of experience dedicated to abdominal radiology, respectively) independently reviewed images for qualitative analysis. Both were blinded to each patient’s clinical data. For every patient, each investigator determined suspicion for malignancy, invasion of common bile duct (CBD) or duodenum, involvement of uncinate process or neck, presence of peripancreatic fat invasion, retroperitoneal margin status, presence of necrosis (centrally located cystic change of the tumor with irregular internal margin), hyperenhancement of tumor (equal or brighter than pancreatic parenchyma in the hepatic venous phase (HVP)), worrisome finding for invasion of major vessels (celiac artery, superior mesenteric artery (SMA), gastroduodenal artery (GDA), portal vein (PV), and superior mesenteric vein (SMV)) according to the National Comprehensive Cancer Network (NCCN) criteria [20], presence of LN suspicious for metastasis in regional and retroperitoneal space (short diameter > 8 mm for regional and > 10 mm for retroperitoneal station, round, irregular or necrotic change), degree of remnant pancreatic parenchymal atrophy (none to mild versus moderate to severe), presence of underlying chronic pancreatitis suspected on CT (at least two of the following features; pancreatic parenchymal calcification, pancreatolith, irregular dilatation of main pancreatic duct (MPD), and diffuse pancreatic atrophy), presence of acute pancreatitis or pseudocyst, and overall radiologists’ conclusion for resectability (resectable or borderline resectable). A pseudocyst was considered over a retention cyst when there was inflammatory change around the lesion or lesional wall thickening [21]. After individual review, the two reviewers met to reach a consensus on discordant results.

Quantitative analysis was performed by one radiologist (S.H.H.). The longest diameter of the tumor and the MPD were measured. The size of the tumor was measured at either AP or PVP, at whichever the tumor was most clearly demarcated. Finally, the TNM stage was calculated automatically according to the seventh and eighth editions of the American Joint Committee on Cancer (AJCC).

Based on prospective records of clinical recurrence, one radiologist (S.H.H.) retrospectively reviewed each patient’s CTs and determined the actual time of tumor recurrence.

### 2.4. Statistical Analysis

Univariate and multivariate Cox regression analyses were performed to identify preoperative clinical information and CT findings associated with tumor recurrence and OS. Factors which achieved statistical significance in the univariate Cox model or were considered as important for oncologic outcome by investigators were included in the multivariate Cox analysis. A prediction model was derived from the multivariate Cox analysis, and a nomogram was constructed using the package survival and rms of R version 3.1.3 (http://www.r-project.org). The serum CA19-9 was dichotomized at 34 U/mL, which was chosen by the log-rank test using the Contal and O’Quigley method [22]. Harrell’s C-index was measured to evaluate the discriminative ability of the final model. Calibration performance was evaluated by calibration plots.

Internal validation was performed by measuring Harrell’s C-index and incremental area under curve (iAUC) using 200 bootstrapped resamples. Kaplan-Meier curves of the two risk groups based on the predicted probability of event according to the proposed nomogram were compared using the log-rank test. A subgroup analysis was performed on the 82 patients with resectable pancreatic cancer. To identify clinical and pathologic characteristics according to risk, the Mann-Whitney U test and Chi-square test were performed. Continuous variables were described as medians ± standard deviations, and categorical variables were described as frequencies (%). Two-sided *P* values < 0.05 were considered statistically significant. All statistical analyses were performed using SAS version 9.4 (SAS Institute, Cary, NC, USA).

## 3. Results

### 3.1. Patient Demographics 

Of 136 included patients, 77 (56.6%) were male and 59 (43.4%) were female. The median age was 66 (range: 41–81) years and median BMI was 22.7 (range: 15.9–32.3). Of all patients, 77.9% (106 of 136) were symptomatic. Median preoperative serum CA 19-9 was 119.7 U/mL (range: 0.1–13,800.0) and median serum total bilirubin was 2.3 mg/dL (range: 0.1–38.3). In 74.3% (101 of 136) of patients, serum CA 19-9 was above 34 U/mL. In preoperative CT, the median maximal diameter of the tumor was 23.6 mm (range: 11.8–50.2). Eighty-two (60.3%) patients had resectable tumors and 54 (39.7%) had borderline resectable tumors. Most patients (92.6%, 126 of 136) underwent pylorus-preserving pancreaticoduodenectomy and the remaining 10 patients underwent Whipple’s operation. Combined vascular resection was performed in 36 (26.5%) patients. In postoperative pathologic examination, the median maximum diameter of the cancer was 25.0 mm (range: 12–70). One hundred and nine patients (80.1%) were moderately differentiated. Lymphovascular and perineural invasion were observed in 65 (47.8 %) and 113 (83.1%) patients, respectively. Lymph node metastasis was found in 87 (64.0%) patients. Resection margin was involved in 23 (16.9%). Most patients (80.1%, 109 of 136) received postoperative adjuvant chemotherapy. The median duration of follow-up was 527.5 days (range: 106–2710) after surgery. During follow-up, 95 (69.9%) patients developed tumor recurrence and 73 (53.7%) died (Table 1).

### 3.2. Correlation between Preoperative Clinically Detectable Parameters and Long-Term Oncologic Outcomes Following Pancreatectomy

In the univariate Cox regression for DFS, symptoms at diagnosis (*P* = 0.006), preoperative serum CA19-9 (*P* = 0.053), serum total bilirubin (*P* = 0.001), tumor size on CT (*P* = 0.045), CBD invasion on CT (*P* = 0.014), PV or SMV invasion on CT (*P* = 0.011), and borderline resectable tumors on CT (*P* = 0.028) were statistically significant preoperative parameters. The preoperative CT findings of uncinate process involvement (*P* = 0.066) and necrosis (*P* = 0.094) showed similar tendency without statistical significance. Multivariate analysis identified symptoms at diagnosis (Hazard ratio (HR), 2.01; 95% CI, 1.12–3.62; *P* = 0.020), PV or SMV invasion on CT (HR, 2.07; 95% CI, 1.35–3.19; *P* = 0.001), and associated pancreatitis or pseudocysts (HR, 0.51; 95% CI, 0.30–0.86; *P* = 0.013) as independent prognostic factors for tumor recurrence. Preoperative serum CA19-9 (HR, 1.63; 95% CI, 0.97–2.72, *P* = 0.065) and necrosis on CT (HR, 1.64; 95% CI, 0.97–2.79, *P* = 0.066) showed marginal significance (Table S1). 

In the univariate Cox regression for OS, symptoms at diagnosis (*P* = 0.037), preoperative serum CA19-9 (*P* = 0.007), necrosis on CT (*P* = 0.001), and PV invasion on CT (*P* = 0.011) were statistically significant preoperative parameters. Serum total bilirubin (*P* = 0.074), presence of suspected metastatic retroperitoneal LN on CT (*P* = 0.081), and moderate to severe degree of pancreatic parenchymal atrophy on CT (*P* = 0.093) showed marginal significance. Subsequent multivariate analyses identified preoperative serum CA19-9 (HR, 2.29; 95% CI, 1.19–4.39, *P* = 0.013), necrosis on CT (HR, 2.42; 95% CI, 1.39–4.21, *P* = 0.002) and associated pancreatitis or pseudocysts (HR, 0.54; 95% CI, 0.30–0.98; *P* = 0.041) as independent prognostic factors for overall survival. Symptoms at diagnosis (HR, 1.68; 95% CI, 0.88-3.22; *P* = 0.115), PV or SMV invasion on CT (HR, 1.53; 95% CI, 0.92–2.54; *P* = 0.099), and presence of suspicious regional LN on CT (HR, 1.67; 95% CI, 0.99–2.81; *P* = 0.055) showed marginal significance (Table S2).

### 3.3. Subgroup Analysis of the 82 Patients with Resectable Pancreatic Cancer

In the univariate Cox regression for DFS, symptoms at diagnosis (*P* = 0.013), preoperative serum CA19-9 (*P* = 0.027), serum total bilirubin (*P* = 0.009), and tumor size on CT (*P* = 0.042) were statistically significant preoperative parameters. The preoperative CT findings of CBD invasion on CT (*P* = 0.065), duodenal invasion (*P* = 0.105), necrosis (*P* = 0.056), and associated pancreatitis or pseudocyst on CT (*P* = 0.054) showed similar tendency without statistical significance (Table S3). In multivariate analysis, symptoms at diagnosis (*P* = 0.027), preoperative serum CA 19-9 ≥ 34 U/mL (*P* = 0.043), and associated pancreatitis or pseudocyst on CT (*P* = 0.018) were significant prognostic factors, whereas necrosis on CT (*P* = 0.325), which was marginally significant in all cases, was not (Table S4). 

In the univariate Cox regression for OS, symptoms at diagnosis (*P* = 0.027), preoperative serum CA19-9 (*P* = 0.027), serum total bilirubin (*P* = 0.008), serum albumin (*P* = 0.017), and necrosis on CT (*P* = 0.003) were statistically significant preoperative parameters. Presence of retroperitoneal LN on CT (*P* = 0.071) showed marginal significance (Table S5). In univariate analysis, preoperative serum CA19-9 (*P* = 0.021) and necrosis on CT (*P* = 0.045) were significant prognostic factors, whereas associated pancreatitis or pseudocyst on CT (*P* = 0.131), which was significant in all cases, was not (Table S4).

### 3.4. Developing a Preoperative Clinical and Imaging-Based Nomogram to Predict DFS and OS 

**Figure 1:**
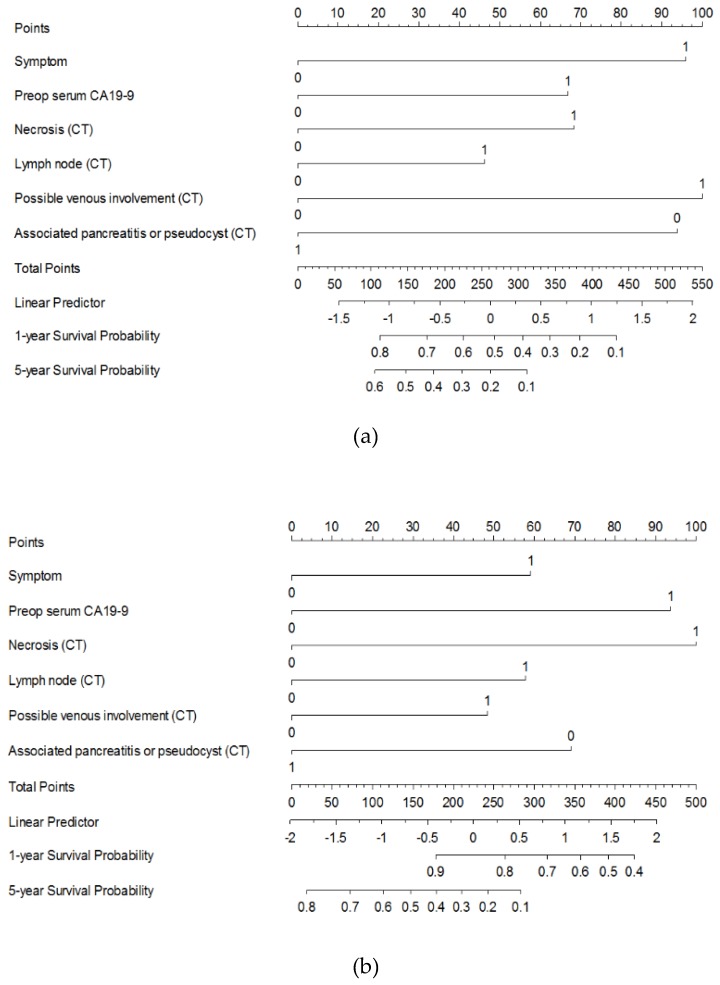
Nomogram predicts probability of (**a**) disease-free survival and (**b**) overall survival one year and five years after curative-intent surgery for radiologically resectable or borderline resectable pancreatic head cancer. The points of each predictor found on the uppermost point scale were added up and the total sum projected on the bottom point scale indicates the probability of (**a**) disease-free and (**b**) overall survival for each time point. The serum CA 19-9 was dichotomized at 34 U/mL by the log-rank test; CA 19-9, carbohydrate antigen 19-9; CT, computed tomography; 0, Negative; 1, Positive.

**Figure 2:**
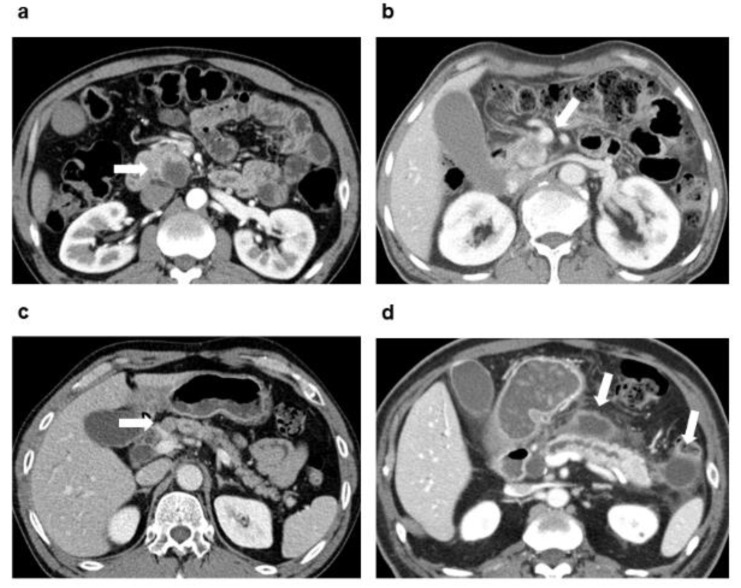
Four preoperative imaging characteristics included in the nomogram to predict one- and five-year DFS and OS in patients with pancreatic head cancer who underwent curative-intent surgery without neoadjuvant therapy. Presence of (**a**) necrosis (centrally located cystic change of the tumor with irregular internal margin), (**b**) worrisome finding for invasion of PV or SMV according to the NCCN guidelines, (**c**) suspected metastatic regional lymph node (short diameter > 8 mm, round, irregular or necrotic change), and (**d**) associated pancreatitis or pseudocyst on CT. DFS, disease-free survival; OS, overall survival; PV, portal vein; SMV, superior mesenteric vein; NCCN, National Comprehensive Cancer Network; CT, computed tomography.

Based on the results of multivariate analysis, a nomogram to predict one- and five- year DFS and OS was derived (Figure 1). The nomogram included six preoperative parameters: Presence of symptoms at diagnosis, preoperative serum CA 19-9 dichotomized at 34 U/mL, necrosis on CT, PV or SMV invasion on CT, presence of suspected metastatic regional LN on CT, and associated pancreatitis or pseudocyst on CT (Figure 2). 

### 3.5. Calibration and Internal Validation

**Figure 3:**
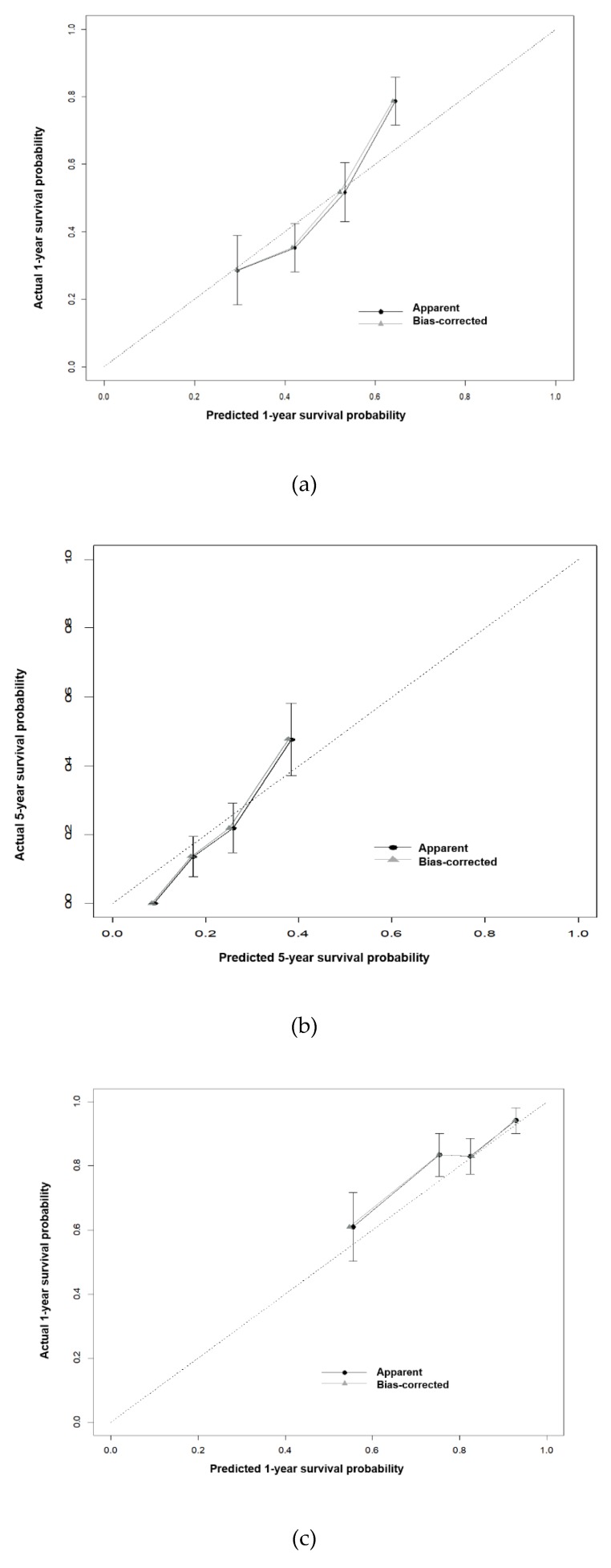

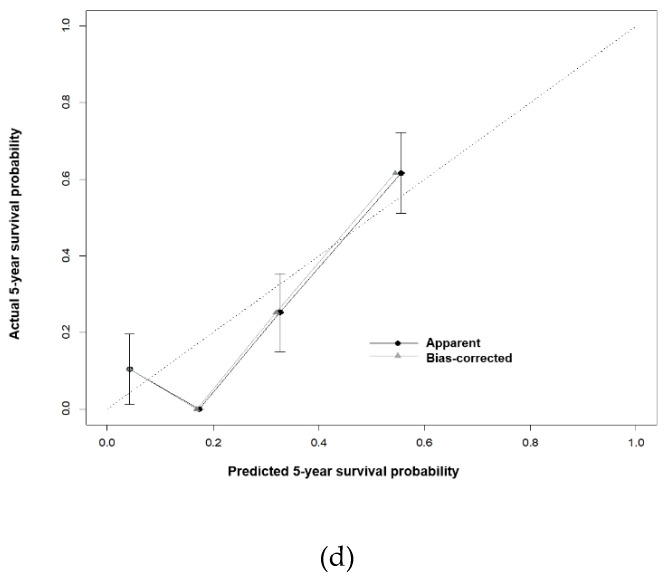
Calibration plots comparing predicted and actual one- and five-year (**a**,**b**) disease-free survival and (**c**,**d**) overall survival.

The model performance, quantified by Harrell’s c-statistics, was 0.6496 (standard error (SE) = 0.0325) for DFS and 0.6746 (SE = 0.0384) for OS (Table 2). Calibration plots showed good agreement between actual and predicted probability for both DFS and OS (Figure 3). 

Internal validation performed using 200 bootstrap resampling revealed that Harrell’s C-index and iAUC of the suggested nomogram was 0.564 (95% CI, 0.527–0.608) and 0.556 (95% CI, 0.505–0.604) for DFS, and 0.579 (95% CI, 0.525–0.633) and 0.579 (95% CI, 0.536–0.626) for OS, respectively.

The ‘apparent’ curve was calculated directly from the dataset. The ‘bias-corrected’ curve was adjusted by bootstrap with 200 resamples.

### 3.6. Risk Stratification According to the Proposed Nomogram to Predict Oncologic Outcomes in Resected Pancreatic Cancer Without Neoadjuvant Treatment

**Figure 4:**
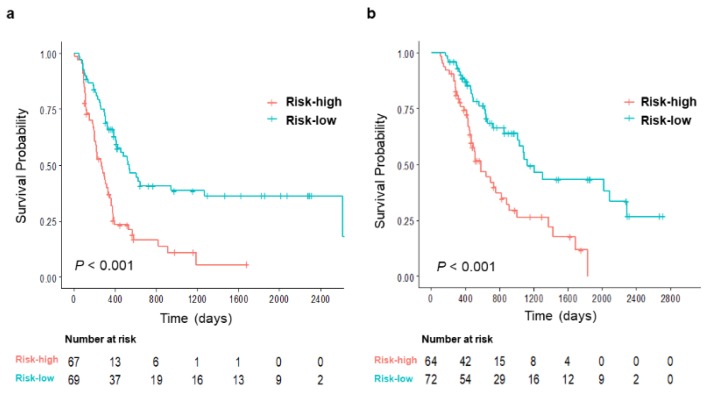
Risk stratification according to the calculated probability of five-year (**a**) disease-free survival and (**b**) overall survival.

Based on the 50% probability of five-year survival calculated from the nomogram, patients were divided into low- and high-risk groups. There were statistically significant differences between risk groups regarding both DFS and OS (both *P* < 0.001; Figure 4). For predicting DFS, there were significant differences in preoperative serum CA 19-9, tumor size, and presence of LN metastasis revealed in postoperative histopathologic examinations between the two groups. Risk groups for predicting OS also showed similar tendency, but only preoperative serum CA 19-9 reached statistical significance (Table 3). There were no significant differences in the proportion of patients receiving adjuvant chemotherapy (*P* = 0.465 for RFS, *P* = 0.156 for OS) or the type of chemotherapy regimen (*P* = 0.607 for DFS, *P* = 0.882 for OS).

High- and low- risk groups were dichotomized at 50% of the nomogram-based calculated probability of recurrence.

### 3.7. Developing a Preoperative Clinical and Imaging-Based Nomogram for Patients Resectable Pancreatic Cancer

Based on the results of multivariate analysis of 82 patients with resectable pancreatic cancer, a nomogram to predict one- and five- year DFS and OS was derived (Figure S1). The nomogram included the same parameters as those of entire study population. The model performance, quantified by Harrell’s c-statistics, was 0.716 (SE = 0.040) for DFS and 0.668 (SE = 0.039) for OS (Table S4). Calibration plots showed good agreement between actual and predicted probability for both DFS and OS (Figure S2). Internal validation performed using 200 bootstrap resampling revealed that Harrell’s C-index and iAUC of the suggested nomogram was 0.695 (95% CI, 0.622-0.763) and 0.693 (95% CI, 0.619–0.755) for DFS, and 0.722 (95% CI, 0.646–0.796) and 0.713 (95% CI, 0.641–0.794) for OS, respectively. Based on the 50% probability of five-year survival calculated from the nomogram, patients were divided into low- and high-risk groups. There were statistically significant differences between risk groups regarding both DFS and OS (both *P* < 0.001; Figure S3).

## 4. Discussion

In the present study, a nomogram based on preoperative clinical and radiologic parameters was derived to predict postoperative oncologic outcomes of patients with pancreatic head cancer who underwent curative-intent surgery without neoadjuvant therapy. It included symptoms at diagnosis, preoperative serum CA 19-9 dichotomized at 34 U/mL, and four CT features (necrosis, possible venous invasion, suspected metastatic regional LN, and associated pancreatitis or pseudocyst) to predict one- and five-year DFS and OS. Kaplan-Meier analysis revealed that the two risk groups defined by the proposed nomogram had clearly different survival rates, a potential advantage of the nomogram (*P* < 0.001).

Brennan et al. proposed a nomogram to predict postoperative disease-specific survival of patients undergoing resection of pancreatic cancer [18], which was externally validated for large populations in the U.S. [23] and Europe [24,25]. Its concordance index was 0.64 and 0.61–0.62 in the external validation [23,24]. Brennan’s nomogram seems to show better performance than our nomogram (iAUC for OS, 0.579 (95% CI, 0.536–0.626)). However, they included all patients with resected pancreatic cancer regardless of tumor location, although a majority (89.4% (496 of 555)) of the cancers were found in the head. Moreover, along with clinical information (age, sex, presence of back pain or weight loss), postoperative information, such as surgical extent and pathologic status, were the main factors in their nomogram. Recently, a Chinese group proposed a nomogram that considered both clinical and preoperative CT findings to predict OS in patients with resectable pancreatic head cancer [26]. However, they only included patients with borderline resectable cancer with suspected venous invasion on CT. No study considering various preoperative CT findings along with clinical information to predict prognosis has been reported so far for patients with radiologically resectable and borderline resectable pancreatic head cancer. Furthermore, we found the same trend when analyzing patients with borderline resectable or resectable pancreatic cancer and when analyzing only patients with resectiable pancreatic cancer.

Tumor necrosis caused by hypoxia is generally regarded as a poor prognostic factor and was reported as an independent prognostic factor of DFS in a past pathologic study [27]. In our study, necrosis on CT was observed in 19.9% (27 of 136) patients, and median serum CA 19-9 was marginally higher (1297.76 (SE, 457.12) vs. 428.10 (SE, 138.94); *P* = 0.078) in patients with necrosis on CT than those without.

Although pathologic node-positivity is an important predictor of five-year OS [28], there is an obvious limitation in preoperative diagnosis of LN metastasis with imaging [29]. In our study, when a short diameter of 8 mm was used as a cutoff for regional LN metastasis on CT, the diagnostic sensitivity and specificity was 40.2% (35 of 87) and 81.6% (40 of 49), respectively. The sensitivity was higher than previous studies using 10 mm as a cutoff (14%) with comparable specificity. LN metastasis seems to be a poor prognostic factor for tumor recurrence and OS, although statistically significant only for recurrence. Vyas et al. reported the presence of LN 10 mm or larger on CT regardless of pathologic LN status as a prognostic factor [30]. As the systemic inflammatory response syndrome with high preoperative c-reactive protein levels has been reported as a poor prognostic factor for pancreatic cancer [31], they suggested that an enlarged LN might be associated with systemic inflammation. Shen et al. also reported node-positivity on CT as a prognostic factor in patients with borderline resectable pancreatic head cancer, although they did not clarify the definition of positive LN on CT [26]. In our study, the LN positivity assessed by CT was not a significant factor in predicting survival when analyzed in patients only with resectable pancreatic cancer, as opposed to patients with borderline or resectable pancreatic cancer. No evidence was found in our study for changes in the upfront surgical policy for patients with resectable pancreatic cancer with suspected local LN metastases in CT but without other risk factors.

In our study, the presence of pancreatitis or pseudocyst on CT was a good prognostic factor for both DFS and OS. One-year OS has been reported to be higher in patients with pancreatic cancer associated with acute pancreatitis than all patients [32]. Early-onset symptoms enabling early diagnosis may be a reason for the higher OS. However, the presence of symptoms is a poor prognostic factor. Of all patients, 77.9% (106 of 136) were symptomatic (jaundice (*n* = 65), abdominal pain (*n* = 48), weight loss/anorexia (*n* = 22), nausea and/or vomiting (*n* = 4)) with higher serum CA 19-9 (716.33 (SE, 184.97) vs. 192.4 (SE, 59.26); *P* = 0.008) and larger tumor size on surgical pathology (27.1 mm (SE, 0.82) vs. 23.5 (SE, 1.47); *P* =0.044) than asymptomatic patients. Considering that our population was entirely composed of patients with pancreatic head cancer, we can presume that the larger the tumor burden is, the more likely it is to be symptomatic.

There are limitations in this study. First, all patients were allocated to the derivative set because of the small population size, meaning that the validation method was limited to bootstrapping. The generalizability of the nomogram should be externally validated with an independent population before being applied to clinical practice. There is a possibility that critical variables infrequently detected might have been neglected because of the small population size. In our study, venous invasion on CT was a prognostic factor showing marginal statistical significance, whereas arterial involvement and overall resectability on CT were not independent prognostic factors. Arterial involvement is considered a more important prognostic factor than venous invasion in patients with borderline resectable pancreatic cancer [33]. However, there were only four borderline resectable tumors with arterial invasion, of which two were suspected for venous invasion. The low frequency of positive findings might explain why this factor was not included in the model. In addition, it might be difficult to differentiate pseudocysts from other cystic lesions such as retention cysts. To reduce misdiagnosis, cystic lesions were evaluated by an experienced radiologist and they were regarded positive only when there was no need to differentiate them from other diagnosis.

In conclusion, the present nomogram based on preoperatively detectable clinical and radiologic parameters can potentially help clinicians decide management plans for pancreatic head cancers which are radiologically resectable and candidates for curative-intent surgery without neoadjuvant therapy.

## Figures and Tables

**Table 1:** Baseline characteristics of the 136 included patients.

Characteristics	Total
**Clinical information**	
Age (years)	66 (41–81)
Gender	
Male	77 (56.6%)
Female	59 (43.4%)
BMI (kg/m^2^)	22.7 (15.9–32.3)
PNI	48.2 (30.4–60.1)
Symptoms at diagnosis ^†^	106 (77.9%)
Preoperative serum CA19-9 (continuous) (U/mL)	119.7 (0.1–13,800.0)
Serum total bilirubin at diagnosis (mg/dL)	2.3 (0.1–38.3)
Serum albumin at diagnosis (mg/dL)	4.0 (2.7–5.0)
Type of surgery	
Whipple’s operation	10 (7.4 %)
PPPD	126 (92.6%)
Combined vascular resection	36 (26.5%)
Adjuvant chemotherapy	109 (80.1%)
**CT findings**	
Size (mm)	23.6 (11.8–50.2)
Radiologists’ conclusion for resectability	
Resectable	82 (60.3%)
Borderline resectable	54 (39.7%)
**Pathologic findings**	
Size (mm)	25.0 (12–70)
Differentiation	
Well	15 (11.0%)
Moderate	109 (80.1%)
Poorly	12 (8.8%)
Lymphovascular invasion	65 (47.8%)
Perineural invasion	113 (83.1%)
Lymph node metastasis	87 (64.0%)
Resection margin	
Negative	113 (83.1%)
Positive	23 (16.9%)

^†^ Jaundice (*n* = 65), abdominal pain (*n* = 48), weight loss/anorexia (*n* = 22), nausea and/or vomiting (*n* = 4); one patient reported multiple symptoms. Continuous variables are displayed as medians, with ranges in parentheses; BMI, body mass index; CA 19-9, carbohydrate antigen 19-9; PPPD, pylorus-preserving pancreaticoduodenectomy; CT, computed tomography.

**Table 2:** Preoperative parameters to predict disease-free survival and overall survival of patients with resectable pancreatic cancer.

Parameters	Disease-Free Survival			Overall Survival		
	HR	95% CI	*P* Value	HR	95% CI	*P* Value
Symptoms at diagnosis	2.01	1.12–3.62	0.020	1.68	0.88–3.22	0.115
Preoperative serum CA 19-9 ≥ 34 U/mL	1.63	0.97–2.72	0.065	2.29	1.19–4.39	0.013
Necrosis on CT	1.64	0.97–2.79	0.066	2.42	1.39–4.21	0.002
PV or SMV invasion on CT	1.4	0.89–2.21	0.150	1.67	0.99–2.81	0.055
Regional LN suspicious for metastasis on CT	2.07	1.35–3.19	0.001	1.53	0.92–2.54	0.099
Associated pancreatitis or pseudocyst on CT	0.51	0.30–0.86	0.013	0.54	0.30–0.98	0.041
Harrell’s c-statistics	0.6496 (0.0325)			0.6746 (0.0384)		

Numbers in parentheses are standard errors. HR, hazard ratio; CI, confidence interval; CA 19-9, carbohydrate antigen 19-9; CT, computed tomography; PV, portal vein; SMV, superior mesenteric vein; LN, lymph node; AIC, Akaike information criterion.

**Table 3:** Clinical and pathologic characteristics of proposed nomogram-based risk groups. CA 19-9, carbohydrate antigen 19-9; CT, computed tomography; AJCC, American Joint Committee on Cancer.

Chracteristics	Disease-Free Survival			Overall Survival		
	Low-Risk (*n* = 69)	High-Risk (*n* = 67)	*P* Value	Low-Risk (*n* = 72)	High-Risk (*n* = 64)	*P* Value
**Clinical information**						
Age, year (Q1–Q3)	66 (62–71)	64 (55–71)	0.237	66 (58.5–71)	65.5 (59–71)	0.843
Gender			0.712			0.791
Male	38 (55.1%)	39 (58.2%)		40 (55.6%)	37 (57.8%)	
Female	31 (44.9%)	28 (41.8%)		32 (44.4%)	27 (42.2%)	
Preoperative serum CA 19-9(continuous), U/mL (Q1–Q3)	56.0 (26.2–28.6)	203.9 (49.2–734.8)	0.003	54.0 (19.9–212.6)	295.0 (97.6–772.0)	<0.001
Adjuvant chemotherapy	57(82.6%)	52(77.6%)	0.4652	61 (84.7%)	48 (75.0%)	0.156
Gemcitabine-based	42 (53.8%)	36 (46.2%)	0.607	44 (56.4%)	34 (46.6%)	0.882
Other	15 (48.4%)	16 (51.6%)		17 (54.8%)	14 (45.2%)	
**Pathologic findings**						
Size, mm (Q1–Q3)	23 (20–30)	26 (22–32)	0.019	23.5 (20–30)	25.5 (22–30)	0.057
Peripancreatic fat invasion	68 (98.6%)	67 (100.0 %)	>0.999	71 (98.6%)	64 (100.0%)	>0.999
Differentiation			0.741			0.844
Well	7 (10.1%)	8 (11.9%)		7 (9.7%)	8 (12.5%)	
Moderate	57 (82.6%)	52 (77.6%)		59 (82.0%)	50 (78.1%)	
Poorly	5 (7.2%)	7 (10.4%)		6 (8.3%)	6 (9.4%)	
Lymphovascular invasion	31 (45.6%)	30 (44.8%)	0.925	34 (47.9%)	29 (45.3%)	0.765
Perineural invasion	56 (82.4%)	57(85.1%)	0.669	60 (84.5%)	53 (82.8%)	0.790
Lymph node metastasis	38 (55.1%)	49 (73.1%)	0.028	41 (56.9%)	46 (71.9%)	0.070
Lymph node ratio (Q1–Q3)	0.04 (0–0.15)	0.06 (0–0.18)	0.143	0.06 (0–0.15)	0.06 (0–0.18)	0.306
Resection margin positive	9 (13.0%)	14 (20.9%)	0.240	12 (16.7%)	11 (17.2%)	0.503

## Supplementary Materials

The following are available online at https://www.mdpi.com/2077-0383/8/10/1749/s1.
