# Supplementary material for: Preoperative Clinical and Computed Tomography (CT)-Based Nomogram to Predict Oncologic Outcomes in Patients with Pancreatic Head Cancer Resected with Curative Intent: A Retrospective Study
Source: J Clin Med. 2019 Oct 21;8(10):1749. doi: 10.3390/jcm8101749 (PMC6833079; doi:10.3390/jcm8101749)
Supplement: Supplementary file 1 [file jcm-08-01749-s001.pdf]

**Table S1.** Univariate analysis of preoperative clinical and computed tomography findings in patients with resectable and borderline pancreatic cancer to predict tumor recurrence.

| Variables | HR | 95% CI | *P* value |
|---|---|---|---|
| **Clinical information** | | | |
| Age, year | 0.996 | 0.97-1.02 | 0.767 |
| Gender | | | |
| Male† | | | |
| Female | 0.82 | 0.54-1.24 | 0.343 |
| BMI | 1.01 | 0.95-1.08 | 0.680 |
| PNI | 0.98 | 0.95-1.02 | 0.327 |
| Symptoms at diagnosis | 2.23 | 1.26-3.93 | 0.006 |
| Preoperative serum CA19-9 (continuous) (U/mL) | 1 | 1.00-1.00 | 0.021 |
| Preoperative serum CA19-9 ≥ 34 U/mL | 1.6 | 0.99-2.59 | 0.053 |
| Serum total bilirubin at diagnosis (mg/dL) | 1.05 | 1.02-1.08 | 0.001 |
| Serum albumin at diagnosis | 0.72 | 0.45-1.13 | 0.145 |
| Type of surgery | | | |
| Whipple's operation† | | | |
| PPPD | 1.81 | 0.73-4.45 | 0.198 |
| **CT findings** | | | |
| Tumor size (maximum diameter, mm) | 1.03 | 1.00-1.05 | 0.045 |
| CBD invasion | 1.82 | 1.13-2.94 | 0.014 |
| Duodenal invasion | 1.12 | 0.74-1.68 | 0.598 |
| Uncinate process involvement | 1.47 | 0.97-2.21 | 0.066 |
| Neck involvement | 0.85 | 0.21-3.46 | 0.822 |
| Peripancreatic fat invasion, | 1.02 | 0.57-1.84 | 0.949 |
| Positive retroperitoneal margin | 1.16 | 0.54-2.50 | 0.712 |
| Necrosis | 1.53 | 0.93-2.51 | 0.093 |
| Tumor hyperenhancement | 0.99 | 0.48-2.05 | 0.979 |
| Worrisome finding for invasion of major vessels | | | |
| CA, SMA, or GDA | 0.95 | 0.30-3.00 | 0.927 |
| PV | 2.5 | 1.15-5.47 | 0.021 |
| SMV | 1.53 | 1.01-2.32 | 0.047 |
| PV or SMV | 1.74 | 1.13-2.59 | 0.011 |
| LN suspicious for metastasis | | | |
| Regional | 1.34 | 0.88-2.04 | 0.168 |
| Retroperitoneum | 1.35 | 0.75-2.43 | 0.314 |
| Maximum MPD diameter (mm) | 0.995 | 0.94-1.05 | 0.864 |
| Pancreatic parenchymal atrophy | | | |
| None to mild† | | | |
| Moderate to severe | 0.9 | 0.60-1.36 | 0.627 |
| Chronic pancreatitis in the CT | 2.57 | 0.63-10.51 | 0.188 |
| Associated pancreatitis or pseudocyst | 0.71 | 0.43-1.18 | 0.186 |
| Radiologists' conclusion for resectability | | | |
| Resectable† | | | |
| Borderline resectable | 1.58 | 1.05-2.38 | 0.028 |

† Used as the reference category. "Hyperenhancement" means that the tumor is equal or brighter than pancreatic parenchyma in the hepatic venous phase. Worrisome findings for the invasion of major vessels were based on the National Comprehensive Cancer Network criteria. Regional LN larger than 8mm, retroperitoneal LN larger than 10mm, or any LN with round-shape or necrosis was considered abnormal. Chronic pancreatitis was presumed if the underlying pancreas showed at least two of the following features - pancreatic parenchymal calcification, pancreaticolith, irregular dilatation of MPD, and diffuse pancreatic atrophy. HR, hazard ratio; CI, confidence interval; BMI, body mass index; PNI, prognostic nutritional index; CA 19-9, carbohydrate antigen 19-9; PPPD, pylorus-preserving pancreaticoduodenectomy; LN, lymph node; PNI, perineural invasion; LVI, lymphovascular invasion; CBD, common bile duct; AJCC, American Joint Committee on Cancer; CT, computed

tomography; CA, celiac artery; SMA, superior mesenteric artery; GDA, gastroduodenal artery; PV, portal vein; SMV, superior mesenteric vein; MPD, main pancreatic duct.

**Table S2.** Univariate analysis of preoperative clinical and computed tomography findings in patients with resectable and borderline resectable pancreatic cancer to predict overall survival.

| Variables | HR | 95% CI | *P* value |
|---|---|---|---|
| **Clinical information** | | | |
| Age, year | 1.02 | 0.99-1.05 | 0.298 |
| Gender | | | |
| Male† | | | |
| Female | 0.69 | 0.43-1.10 | 0.121 |
| BMI | 0.97 | 0.91-1.05 | 0.469 |
| PNI | 0.97 | 0.93-1.01 | 0.137 |
| Symptoms at diagnosis | 1.95 | 1.04-3.63 | 0.037 |
| Preoperative serum CA19-9 (continuous) (U/mL) | 1 | 1.00-1.00 | 0.289 |
| Preoperative serum CA19-9 ≥ 34 U/mL | 2.3 | 1.26-4.20 | 0.007 |
| Serum total bilirubin at diagnosis (mg/dL) | 1.035 | 1.00-1.07 | 0.074 |
| Serum albumin at diagnosis | 0.69 | 0.40-1.20 | 0.193 |
| Type of surgery | | | |
| Whipple's operation† | | | |
| PPPD | 1.11 | 0.45-2.76 | 0.820 |
| **CT findings** | | | |
| Tumor size (maximum diameter, mm) | 1.02 | 0.99-1.05 | 0.185 |
| CBD invasion | 1.01 | 0.62-1.64 | 0.965 |
| Duodenal invasion | 0.92 | 0.57-1.48 | 0.728 |
| Uncinate process involvement | 1.11 | 0.69-1.79 | 0.666 |
| Neck involvement | 1.35 | 0.33-5.52 | 0.679 |
| Peripancreatic fat invasion, | 1.09 | 0.57-2.08 | 0.795 |
| Positive retroperitoneal margin | 1.36 | 0.59-3.16 | 0.470 |
| Necrosis | 2.45 | 1.46-4.14 | 0.001 |
| Tumor hyperenhancement | 1.55 | 0.74-3.24 | 0.248 |
| Worrisome finding for invasion of major vessels | | | |
| CA, SMA, or GDA | 0.45 | 0.06-3.27 | 0.433 |
| PV | 2.28 | 1.04-4.98 | 0.040 |
| SMV | 1.07 | 0.65-1.76 | 0.781 |
| PV or SMV | 1.25 | 0.77-2.01 | 0.367 |
| LN suspicious for metastasis | | | |
| Regional | 1.37 | 0.86-2.20 | 0.186 |
| Retroperitoneum | 1.74 | 0.93-3.25 | 0.081 |
| Maximum MPD diameter (mm) | 1.03 | 0.98-1.10 | 0.268 |
| Pancreatic parenchymal atrophy | | | |
| None to mild† | | | |
| Moderate to severe | 1.49 | 0.94-2.37 | 0.093 |
| Chronic pancreatitis in the CT | 2.54 | 0.61-10.47 | 0.199 |
| Associated pancreatitis or pseudocyst | 0.88 | 0.52-1.50 | 0.643 |
| Radiologists' conclusion for resectability | | | |
| Resectable† | | | |
| Borderline resectable | 1.19 | 0.74-1.92 | 0.462 |

† Used as the reference category. "Hyperenhancement" means that the tumor is equal or brighter than pancreatic parenchyma in the hepatic venous phase. Worrisome findings for the invasion of major vessels were based on the National Comprehensive Cancer Network criteria. Regional LN larger than 8mm, retroperitoneal LN larger than 10mm, or any LN with round-shape or necrosis was considered abnormal. Chronic pancreatitis was presumed if the underlying pancreas showed at least two of the following features - pancreatic parenchymal calcification, pancreaticolith, irregular dilatation of MPD, and diffuse pancreatic atrophy. HR, hazard ratio; CI, confidence interval; BMI, body mass index; PNI, prognostic nutritional index; CA 19-9, carbohydrate antigen 19-9; PPPD, pylorus-preserving pancreaticoduodenectomy; LN, lymph node; PNI, perineural invasion; LVI, lymphovascular

invasion; CBD, common bile duct; AJCC, American Joint Committee on Cancer; CT, computed tomography; CA, celiac artery; SMA, superior mesenteric artery; GDA, gastroduodenal artery; PV, portal vein; SMV, superior mesenteric vein; MPD, main pancreatic duct.

**Table S3.** Univariate analysis of preoperative clinical and computed tomography findings in patients with resectable pancreatic cancer to predict tumor recurrence.

| Variables | HR | 95% CI | *P* value |
|---|---|---|---|
| **Clinical information** | | | |
| Age, year | 1.00 | 0.96-1.03 | 0.927 |
| Gender | | | |
| Male† | | | |
| Female | 0.71 | 0.40-1.24 | 0.229 |
| BMI | 0.98 | 0.89-1.07 | 0.587 |
| PNI | 0.98 | 0.93-1.03 | 0.443 |
| Symptoms at diagnosis | 3.21 | 1.27-8.08 | 0.013 |
| Preoperative serum CA19-9 (continuous) (U/mL) | 1.00 | 1.00-1.00 | 0.023 |
| Preoperative serum CA19-9 ≥ 34 U/mL | 2.26 | 1.10-4.65 | 0.027 |
| Serum total bilirubin at diagnosis (mg/dL) | 1.05 | 1.01-1.09 | 0.009 |
| Serum albumin at diagnosis | 0.77 | 0.40-1.45 | 0.414 |
| Type of surgery | | | |
| Whipple's operation† | | | |
| PPPD | 0.99 | 0.31-3.19 | 0.991 |
| **CT findings** | | | |
| Tumor size (maximum diameter, mm) | 1.03 | 1.00-1.07 | 0.042 |
| CBD invasion | 1.98 | 0.96-4.08 | 0.065 |
| Duodenal invasion | 1.58 | 0.91-2.76 | 0.105 |
| Uncinate process involvement | 1.12 | 0.62-2.00 | 0.710 |
| Neck involvement | NA | NA | NA |
| Peripancreatic fat invasion, | 0.89 | 0.43-1.82 | 0.739 |
| Positive retroperitoneal margin | 0.44 | 0.06-3.16 | 0.412 |
| Necrosis | 1.86 | 0.99-3.52 | 0.056 |
| Tumor hyperenhancement | 0.99 | 0.36-2.75 | 0.982 |
| LN suspicious for metastasis | | | |
| Regional | 1.14 | 0.64-2.03 | 0.662 |
| Retroperitoneum | 1.26 | 0.54-2.94 | 0.601 |
| Maximum MPD diameter (mm) | 0.99 | 0.92-1.06 | 0.752 |
| Pancreatic parenchymal atrophy | | | |
| None to mild† | | | |
| Moderate to severe | 0.90 | 0.52-1.56 | 0.705 |
| Chronic pancreatitis in the CT | NA | NA | NA |
| Associated pancreatitis or pseudocyst | 0.40 | 0.16-1.02 | 0.054 |

† Used as the reference category. "Hyperenhancement" means that the tumor is equal or brighter than pancreatic parenchyma in the hepatic venous phase. Worrisome findings for the invasion of major vessels were based on the National Comprehensive Cancer Network criteria. Regional LN larger than 8mm, retroperitoneal LN larger than 10mm, or any LN with round-shape or necrosis was considered abnormal. Chronic pancreatitis was presumed if the underlying pancreas showed at least two of the following features - pancreatic parenchymal calcification, pancreaticolith, irregular dilatation of MPD, and diffuse pancreatic atrophy. HR, hazard ratio; CI, confidence interval; BMI, body mass index; PNI, prognostic nutritional index; CA 19-9, carbohydrate antigen 19-9; PPPD, pylorus-preserving pancreaticoduodenectomy; NA, not applicable; LN, lymph node; PNI, perineural invasion; LVI, lymphovascular invasion; CBD, common bile duct; AJCC, American Joint Committee on Cancer; CT, computed tomography; CA, celiac artery; SMA, superior mesenteric artery; GDA, gastroduodenal artery; PV, portal vein; SMV, superior mesenteric vein; MPD, main pancreatic duct

**Table S4.** Preoperative parameters to predict disease-free survival and overall survival of patients with 82 resectable pancreatic cancers.

| | **Disease-free survival** | | | Overall survival | | |
|---|---|---|---|---|---|---|
| | **HR** | **95% CI** | ***P* value** | HR | 95% CI | *P* value |
| Symptoms at diagnosis | 2.93 | 1.13-7.58 | 0.027 | 2.09 | 0.76-5.71 | 0.151 |
| Preoperative serum CA 19-9 ≥ 34 U/mL | 2.17 | 1.03-4.58 | 0.043 | 3.13 | 1.19-8.21 | 0.021 |
| Necrosis on CT | 1.39 | 0.72-2.70 | 0.325 | 2.08 | 1.02-4.25 | 0.045 |
| PV or SMV invasion on CT | Not applicable | | | | | |
| Regional LN suspicious for metastasis on CT | 1.07 | 0.59-1.95 | 0.520 | 1.61 | 0.84-3.09 | 0.152 |
| Associated pancreatitis or pseudocyst on CT | 0.32 | 0.13-0.82 | 0.018 | 0.52 | 0.22-1.22 | 0.131 |
| Harrell's c-statistics | 0.716 (0.04) | | | 0.668 (0.039) | | |

Numbers in parentheses are standard errors. HR, hazard ratio; CI, confidence interval; CA 19-9, carbohydrate antigen 19-9; CT, computed tomography; PV, portal vein; SMV, superior mesenteric vein; LN, lymph node.

**Table S5.** Univariate and multivariate analysis of preoperative clinical and computed tomography (CT) findings in patients with resectable pancreatic cancer to predict overall survival.

| **Variables** | **HR** | **95% CI** | ***P* value** |
|---|---|---|---|
| **Clinical information** | | | |
| Age, year | 1.03 | 0.99-1.07 | 0.212 |
| Gender | | | |
| Male† | | | |
| Female | 0.68 | 0.37-1.27 | 0.227 |
| BMI | 0.96 | 0.87-1.05 | 0.359 |
| PNI | 0.96 | 0.91-1.02 | 0.160 |
| Symptoms at diagnosis | 2.95 | 1.14-7.67 | 0.027 |
| Preoperative serum CA19-9 (continuous) (U/mL) | 1.00 | 1.00-1.00 | 0.011 |
| Preoperative serum CA19-9 ≥ 34 U/mL | 2.95 | 1.14-7.67 | 0.027 |
| Serum total bilirubin at diagnosis (mg/dL) | 3.55 | 1.39-9.05 | 0.008 |
| Serum albumin at diagnosis | 1.05 | 1.01-1.10 | 0.017 |
| Type of surgery | | | |
| Whipple's operation† | | | |
| PPPD | 0.73 | 0.22-2.36 | 0.596 |
| **CT findings** | | | |
| Tumor size (maximum diameter, mm) | 1.01 | 0.97-1.05 | 0.667 |
| CBD invasion | 1.07 | 0.54-2.13 | 0.837 |
| Duodenal invasion | 1.03 | 0.56-1.88 | 0.925 |
| Uncinate process involvement | 1.22 | 0.63-2.36 | 0.560 |
| Neck involvement | NA | NA | NA |
| Peripancreatic fat invasion, | 1 | 0.46-2.16 | 1 |
| Positive retroperitoneal margin | 1.07 | 0.26-4.48 | 0.923 |
| Necrosis | 2.90 | 1.44-5.84 | 0.003 |
| Tumor hyperenhancement | 1.80 | 0.70-4.61 | 0.224 |
| LN suspicious for metastasis | | | |
| Regional | 1.64 | 0.89-3.02 | 0.114 |
| Retroperitoneum | 2.13 | 0.94-4.82 | 0.071 |
| Maximum MPD diameter (mm) | 1.03 | 0.96-1.12 | 0.375 |
| Pancreatic parenchymal atrophy | | | |
| None to mild† | | | |
| Moderate to severe | 1.61 | 0.87-2.96 | 0.127 |
| Chronic pancreatitis in the CT | NA | NA | NA |
| Associated pancreatitis or pseudocyst | 0.75 | 0.33-1.69 | 0.484 |

† Used as the reference category. “Hyperenhancement” means that the tumor is equal or brighter than pancreatic parenchyma in the hepatic venous phase. Worrisome findings for the invasion of major vessels were based on the National Comprehensive Cancer Network criteria. Regional LN larger than 8mm, retroperitoneal LN larger than 10mm, or any LN with round-shape or necrosis was considered abnormal. Chronic pancreatitis was presumed if the underlying pancreas showed at least two of the following features - pancreatic parenchymal calcification, pancreaticolith, irregular dilatation of MPD, and diffuse pancreatic atrophy. HR, hazard ratio; CI, confidence interval; BMI, body mass index; PNI, prognostic nutritional index; CA 19-9, carbohydrate antigen 19-9; PPPD, pylorus-preserving pancreaticoduodenectomy; LN, lymph node; PNI, perineural invasion; LVI, lymphovascular invasion; CBD, common bile duct; AJCC, American Joint Committee on Cancer; CT, computed tomography; CA, celiac artery; SMA, superior mesenteric artery; GDA, gastroduodenal artery; PV, portal vein; SMV, superior mesenteric vein; MPD, main pancreatic duct

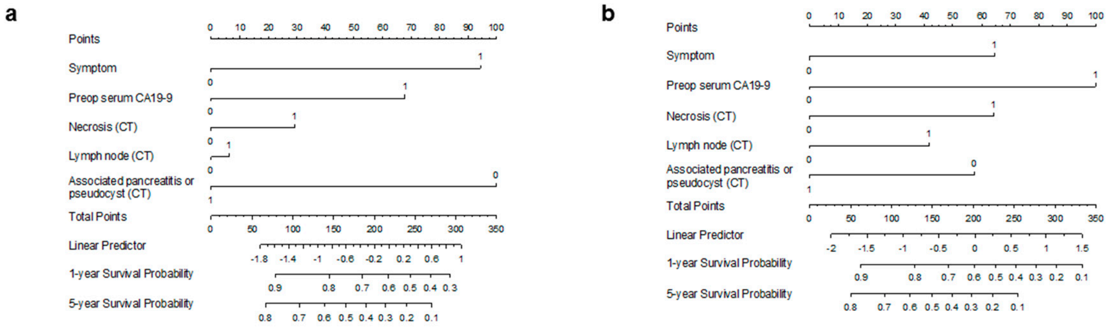


**Figure S1.** Nomogram predicts probability of (a) disease-free survival and (b) overall survival 1 year and 5 years after curative-intent surgery for radiologically resectable pancreatic head cancer. The points of each predictor found on the uppermost point scale are added up and the total sum projected on the bottom point scale indicates the probability of (a) disease-free and (b) overall survival for each time point. The serum CA19-9 was dichotomized at 34 U/mL by the log-rank test; CA 19-9, carbohydrate antigen 19-9; CT, computed tomography; 0, Negative; 1, Positive

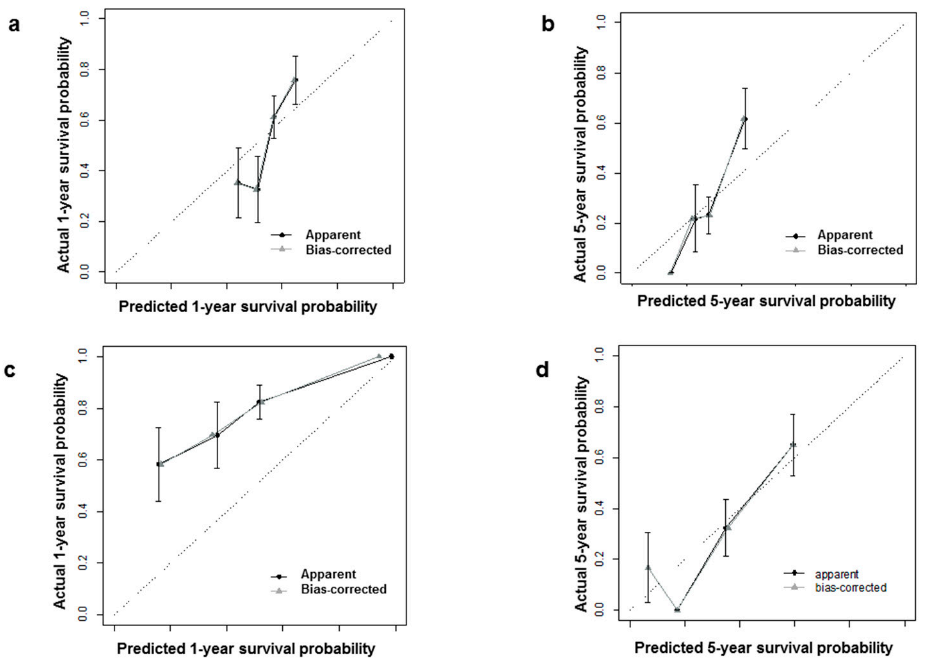


**Figure S2.** Calibration plots comparing predicted and actual 1- and 5-year (a, b) disease-free survival and (c, d) overall survival of patients with radiologically resectable pancreatic head cancer. The 'apparent' curve was calculated directly from the dataset. The 'bias-corrected' curve was adjusted by bootstrap with 200 resamples.

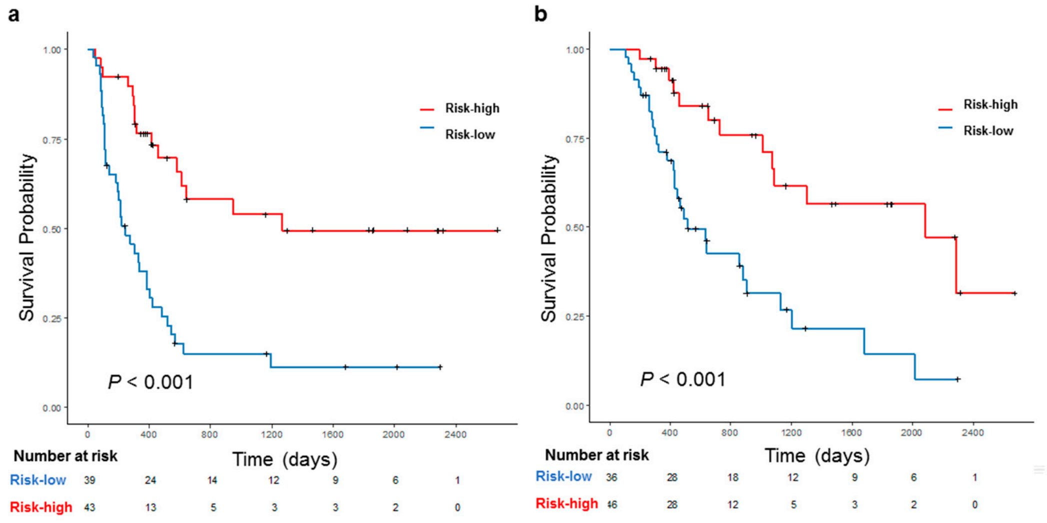


**Figure S3.** Risk stratification according to the calculated probability of 5-year (A) disease-free survival and (B) overall survival of patients with radiologically resectable pancreatic head cancer. High- and low- risk groups are dichotomized at 50% of the nomogram-based calculated probability of recurrence.
